# Moving to the Beat and Singing are Linked in Humans

**DOI:** 10.3389/fnhum.2015.00663

**Published:** 2015-12-18

**Authors:** Simone Dalla Bella, Magdalena Berkowska, Jakub Sowiński

**Affiliations:** ^1^EuroMov, University of MontpellierMontpellier, France; ^2^Institut Universitaire de France (IUF)Paris, France; ^3^International Laboratory for Brain, Music, and Sound Research (BRAMS)Montreal, QC, Canada; ^4^Department of Cognitive Psychology, University of Finance and Management in WarsawWarsaw, Poland

**Keywords:** singing, synchronization, auditory-motor integration, poor-pitch singing, beat deafness

## Abstract

The abilities to sing and to move to the beat of a rhythmic auditory stimulus emerge early during development, and both engage perceptual, motor, and sensorimotor processes. These similarities between singing and synchronization to a beat may be rooted in biology. Patel (2008) has suggested that motor synchronization to auditory rhythms may have emerged during evolution as a byproduct of selection for vocal learning (“vocal learning and synchronization hypothesis”). This view predicts a strong link between vocal performance and synchronization skills in humans. Here, we tested this prediction by asking occasional singers to tap along with auditory pulse trains and to imitate familiar melodies. Both vocal imitation and synchronization skills were measured in terms of accuracy and precision or consistency. Accurate and precise singers tapped more in the vicinity of the pacing stimuli (i.e., they were more accurate) than less accurate and less precise singers. Moreover, accurate singers were more consistent when tapping to the beat. These differences cannot be ascribed to basic motor skills or to motivational factors. Individual differences in terms of singing proficiency and synchronization skills may reflect the variability of a shared sensorimotor translation mechanism.

## Introduction

Singing and dancing are complex activities which are very natural to humans and universally found across societies and cultures (Nettl, 2000; Mithen, 2006, 2009). Carrying a tune and moving to a beat are widespread in the general population, and do not require formal musical training (Dalla Bella et al., 2007; Pfordresher and Brown, 2007; Sowiński and Dalla Bella, 2013). With just few exceptions (i.e., tone-deaf and beat-deaf individuals; Peretz and Hyde, 2003; Dalla Bella et al., 2011; Phillips-Silver et al., 2011; Sowiński and Dalla Bella, 2013; Launay et al., 2014; Tillmann et al., 2015), the majority can carry a tune, when asked to produce a well-known melody or to imitate single pitches, intervals, and novel melodies (e.g., Pfordresher et al., 2010; Berkowska and Dalla Bella, 2013). Similarly they can naturally tap in sync to the beat of simple and complex rhythmic sequences such as a metronome or music (Repp, 2010; Sowiński and Dalla Bella, 2013). Coordinating one own’s behavior to the timing of an external timekeeper in a flexible fashion is typical for humans (McDermott and Hauser, 2005; Patel, 2006) and is an important component of social interaction (Feldman, 2007; Hove and Risen, 2009; Kirschner and Tomasello, 2010; Kleinspehn-Ammerlahn et al., 2011). Both singing and moving to a beat are participatory activities. They are very enjoyable group activities (e.g., during rituals, in the military, in collective entertainment) thought to increase group cohesion and social bonding between group members (Mithen, 2006; Tarr et al., 2014).

The basic skills needed for singing and moving to a beat emerge very soon during development approximately at the same time during the first year of life (Fujii and Schlaug, 2013). A few months after birth, infants produce vowel-like monosyllabic productions called “coos” which act as the precursors of adult singing (Prechtl and Hopkins, 1986; Papoušek, 1996; Masataka, 2007). These first vocal productions emerge spontaneously, for example, by imitation of maternal singing (e.g., Trehub and Trainor, 1999; Trehub and Gudmundsdottir, 2015; Trehub, 2015). After one year of age, toddlers start reproducing recognizable melodies (Ostwald, 1973; Barrett, 2011; Stadler-Elmer, 2012). Starting from these elementary examples of singing skills, vocal performance slowly develops over time thanks to spontaneous practice and early musical tutoring (for a review, see Welch, 2006), leading to accuracy and precision typical of adult singing (Pfordresher et al., 2010; Berkowska and Dalla Bella, 2013). Similarly, there is evidence that infants can extract the beat from auditory patterns. They are sensitive to violations in repetitive timing patterns (i.e., meter; see Hannon and Trehub, 2005; Bergeson and Trehub, 2006; Trehub and Hannon, 2009; Winkler et al., 2009), and can code meter via body movement (Phillips-Silver and Trainor, 2005) like adults do (Phillips-Silver and Trainor, 2007). Early sensitivity to temporal regularities is accompanied by the emergence of spontaneous movement in response to music, more often than to other auditory stimuli (Eerola et al., 2006; Zentner and Eerola, 2010). Building on sensitivity to regular temporal patterns (e.g., the underlying pulse), 2.5-year-old children show first evidence of motor synchronization by adjusting their movement to the beat of an auditory stimulus, in particular when interacting with a social partner (Provasi and Bobin-Bègue, 2003; Kirschner and Tomasello, 2009). Hence, the tie between movement and musical rhythm is likely to be hard-wired and is expressed as early as the first infant-mother interaction (Dissanayake, 2000).

That the precursors of singing and synchronization to a beat emerge approximately at the same time in humans may not be a simple coincidence. Both activities rely on fine-tuned audio-motor coordination which engages similar brain circuitries. Well-coupled perception and action are essential for many activities in everyday life. Thanks to precise mapping of visual, auditory, and tactile/proprioceptive information to coordinated motor patterns we can navigate in the environment (e.g., by walking), interact with others, learn a new language, and perform music in an ensemble (Knoblich and Flach, 2001; Keller, 2008). In all these situations the motor system flexibly adjusts to temporal features of the environment, thus leading to predictive behavior (e.g., anticipatory movement). In particular, fine analysis of sensory feedback allows monitoring of performance and error correction, thereby shaping subsequent actions (for examples in speech and music, see MacKay, 1987; Levelt, 1989; Pfordresher, 2006). Synchronizing movement to a beat relies upon the ability to couple the auditory representation of pacing stimuli (e.g., of a pulse train or music) with a precisely timed motor plan via auditory-motor integration mechanisms (Repp, 2005, 2006; Zatorre et al., 2007; Chen et al., 2009). Mapping auditory-to-motor information is similarly crucial in achieving accurate vocal performance (Pfordresher and Brown, 2007; Dalla Bella et al., 2011; Pfordresher and Mantell, 2014): proficient singing is afforded by online adjustments of the motor output on the basis of auditory feedback.

It is plausible that singing and synchronization to a beat engage common auditory-motor integration or sensorimotor translation mechanisms (Hutchins and Peretz, 2012; Pfordresher et al., 2015a,b). Overarching models including sensorimotor translation have been proposed for explaining both singing (e.g., vocal imitation) and synchronization to a beat (Mates, 1994; Repp, 2006; Dalla Bella et al., 2011; Pfordresher et al., 2015a). Sensorimotor translation involves mapping of a sensory continuum to a related motor continuum thereby allowing online performance monitoring and error correction, when needed, based on auditory feedback, and fostering accurate and precise performance. Interestingly, sensorimotor translation is not specific to singing neither to a given dimension (e.g., pitch or timing), but it is likely to encompass different modalities and probably is not confined to imitative behaviors, as recently suggested by Pfordresher and collaborators (Pfordresher and Mantell, 2014; Pfordresher et al., 2015b). These auditory-motor associations are based on learning the contingencies between sounds and movements (Lahav et al., 2007; Chen et al., 2012), which typically require years of formal training in professional musicians (e.g., Brown et al., 2015; Dalla Bella, 2015b).

Sensorimotor translation has been recently specified for singing by referring to the concept of internal models (Wolpert, 1997; Kawato, 1999). In particular for vocal imitation, it has been proposed that sensorimotor translation is carried out mostly via an inverse model (Pfordresher and Mantell, 2014). This model of vocal gesture based on the perceptual expected outcome of the action would allow singers to match vocal fold tension to the fundamental frequencies of the expected pitch. This possibility has been recently integrated into a more general approach (i.e., the multi-modal imagery association model; Pfordresher et al., 2015b), in which sensorimotor translation in singing is treated as one example of a broader class of mapping schemas which associate motor planning and perception.

Transformation of a sound pattern into a motor pattern has been linked to the activity of the dorsal pathway (Hickok and Poeppel, 2007; Rauschecker and Scott, 2009), engaging dorsal parietal and premotor regions. In particular, sensorimotor translation has been associated with the activity of dorsal premotor cortex (dPMC); this region of the brain is the only motor area bridging auditory areas (i.e., the superior temporal gyrus) and primary motor areas. It is hypothesized that this area underpins integration of sensory and motor information with the goal of carrying out a given action plan (Zatorre et al., 2007; Chen et al., 2009). dPMC is recruited while participants synchronize with sound when features of the pacing rhythm affecting synchronization are manipulated (i.e., accent intensity, and temporal regularity; Chen et al., 2006, 2008). Likewise, manipulating the auditory feedback (e.g., using pitch-shifted feedback) during vocal performance activates the dPMC (Zarate and Zatorre, 2008; Zarate, 2013). Hence, neuronal networks supporting sensorimotor translation in singing and synchronization partly overlap, thus leading to predict a strong association between these two skills.

The aforementioned links between singing and moving to a beat may be partly motivated by common evolutionary roots (Mithen, 2006; Fitch, 2006; Ravignani et al., 2014). One intriguing hypothesis has connected beat-based rhythmic abilities to vocal learning. According to the “vocal learning and synchronization hypothesis” (Patel, 2006, 2008), synchronization to a beat is a by-product of the vocal learning mechanisms that are shared by several bird and mammal species, including humans. The underlying idea is that a strong link between motor and auditory brain areas is a prerequisite for both singing and synchronization. This idea gained particular momentum as non-human animal species (e.g., parrots) were found to display synchronization to a beat akin to human synchronization (Patel et al., 2009a; Schachner et al., 2009; see also Schachner, 2010). There is evidence that sulfur-crested cockatoos (Patel et al., 2009a) and other bird species which are vocal learners (Schachner et al., 2009) can move to a musical beat. When musical excerpts are presented across a wide range of tempos, parrots spontaneously adjust to the beat. Additional compelling evidence of trained synchronization in vocal learner species has been recently provided by Hasegawa et al. (2011), who trained budgerigars to peck at the time of an audio-visual metronome, and analyzed performance making use of advanced circular statistics. Note that this ability significantly differs from rhythmic synchronized displays in the auditory or visual domains observed in many species (e.g., synchronized flashing in fireflies, or rhythmic chorusing in frogs; e.g., Buck, 1988; Greenfield and Schul, 2008; for reviews, see Strogatz, 2003; Ravignani et al., 2014). Indeed, motor synchronization in vocal learners is flexible (i.e., adapting to a wider range of tempos), occur with complex auditory signals, and is cross modal (Patel et al., 2009b). Whether synchronization to a beat extends to non-vocal learners is still an object of debate, and evidence is not conclusive. Sensitivity to rhythm grouping, but not to the downbeat in music, is found in rhesus macaques (Honing et al., 2012). Thus this species would lack the basic perceptual mechanism supporting synchronization to a beat. However, the recent discoveries that a chimpanzee can tap above chance with a 600 ms metronome (Hattori et al., 2013) and that a species of sea lions (California Sea Lion) can be trained to bob their head to the beat of a variety of auditory stimuli (Cook et al., 2013) suggests that beat finding and synchronization may extend to some vocal non-mimics. Thus, there is considerable experimental evidence supporting the hypothesis that motor synchronization to a beat may be underpinned by the neuronal circuitry supporting complex vocal learning.

In sum, theories of sensorimotor translation, the vocal learning and synchronization hypothesis, and neuroimaging evidence point toward a link between singing and synchronization skills in humans. We would expect singing proficiency to covary positively with synchronization skills, an hypothesis which has not been tested so far. This hypothesis was examined in the present study by asking non-musicians to imitate well-known songs, and to tap to the sounds of a metronome. Imitation tasks (or pitch/melody matching tasks) are quite natural and widespread among non-musicians (e.g., Pfordresher and Brown, 2007; Pfordresher et al., 2010) and are usually part of batteries of tasks for assessing singing proficiency in the general population (e.g., the Sung Performance Battery and the Seattle Singing Accuracy Protocol; Berkowska and Dalla Bella, 2013; Demorest et al., 2015). In addition, imitation tasks allow assessing the accuracy and precision of sensorimotor translation (i.e., the match between perceived and produced melodies). Singing proficiency and synchronization to the beat were measured in individuals with variable degrees of singing proficiency (i.e., occasional singers). Singing proficiency was analyzed in terms of accuracy and precision (Pfordresher et al., 2010; Dalla Bella, 2015a) for absolute and relative pitch measures when participants sung with lyrics and with a syllable (like in Pfordresher and Brown, 2007; Dalla Bella and Berkowska, 2009; Pfordresher et al., 2010; Berkowska and Dalla Bella, 2013). These dimensions were treated separately as they were found to dissociate in previous studies, thus possibly recruiting partly separable mechanisms (Dalla Bella and Berkowska, 2009; Berkowska and Dalla Bella, 2013). Comparable measures of synchronization performance (i.e., accuracy and consistency) were obtained in a paced tapping task (like in Sowiński and Dalla Bella, 2013). A relation between accuracy for pitch imitation and motor synchronization, and between precision in pitch imitation and synchronization consistency is expected.

## Materials and Methods

### Participants

Fourty-nine occasional singers (35 females), aged between 19 and 39 years (*M* = 25.1 years) took part in the Experiment for class credit. Most were university students. None of the participants had received formal musical training. Only three participants received private musical lessons for a period between 2 and 6 years. No participants reported past and present hearing problems or articulatory disorders. The study was approved by the Ethics Committee of the University of Finance and Management in Warsaw.

### Materials and Procedure

Participants were asked to imitate familiar melodies, typically well performed by occasional singers (Dalla Bella and Berkowska, 2009; Berkowska and Dalla Bella, 2013) and to perform unpaced and paced tapping tasks.

#### Vocal Imitation Task

Participants imitated three well-known songs with Polish lyrics (Woźny, 1958; Malko, 1992; Piatek, 2005); the full melody (32 notes) of “Brother John”, the first eight bars (25 notes) of the chorus of “Jingle Bells”, and the first four bars (20 notes) of “Sto lat” (i.e., a familiar Polish melody typically sung at birthdays). People without musical or vocal training are typically good at this task which is easier than imitating short novel melodies (Dalla Bella et al., 2007; Berkowska and Dalla Bella, 2013). Notes in each melody were pure tones manipulated so as to have a quasi-vocal smooth onset and decay, as done in previous studies (e.g., Berkowska and Dalla Bella, 2013). Melodies were imitated with lyrics (*Lyrics* condition), and in a separate condition on the syllable /la/ (*Syllable* condition). The Syllable condition was aimed at testing singing proficiency while limiting memory demands for lyrics (Berkowska and Dalla Bella, 2009, 2013). A metronome sounded four beats prior to melody presentation (with Brother John, 96 beats/min, Inter-Beat-Interval, IBI = 625 ms; Jingle Bells, 125 beats/min, IBI = 480 ms; Sto lat, 80 beats/min, IBI = 750 ms); the melody was then presented twice together with the metronome. Finally, the metronome was turned off and participants imitated the melody immediately afterwards as accurately as possible. The melody was presented within the vocal range of individual participants. Moreover, written lyrics were made available to participants during the task.

The recording was preceded by a 10-min warm-up session in which participants sang three well-known Polish songs (Pieski małe dwa, Szła dzieweczka, and Wlazł kotek). Participants’ vocal range was estimated prior to the recording with an adaptive automated procedure (Berkowska and Dalla Bella, 2013). The task and the procedure for computing the vocal range were implemented in Matlab 7.1. Stimuli were presented over Sennheiser eH2270 headphones at a comfortable level. Vocal performance was recorded with a Shure SM58 microphone on a Fostex D2424LV digital recorder (sampling frequency = 44.1 KHz) and subsequently dumped onto an IBM-compatible computer using Audition Software for further analyses. The task lasted approximately 1 h.

#### Tapping Tasks

The participants performed a *Synchronization Task* and a *Spontaneous tapping Task*. In the Synchronization Task (e.g., Repp, 2005, 2006), they tapped with their dominant hand to a sequence of 35 tones (duration of each tone = 30 ms) presented with a 600-ms Inter-Onset-Interval (IOI). The sequence was repeated three times. In the Spontaneous tapping Task (e.g., Fraisse, 1956; Drake et al., 2000), they were asked to tap with their dominant hand for 1 min in a regular fashion without pacing stimuli, and at the rate which seemed most natural to them. The two tasks were performed once before and once after the vocal imitation task (overall, two trials for the Spontaneous tapping Task, and six trials for the Synchronization Task). The stimuli were presented over Sennheiser eH2270 headphones at a comfortable intensity level. Motor responses were recorded with a tapping pad with 1 ms accuracy. The tapping pad provided auditory feedback at the time of the tap. The experiment was run on Presentation software (version 9.90; Neurobehavioral Systems, Inc.) using a IBM-compatible computer. The tapping tasks lasted 15 min in total.

## Analyses

### Vocal Performance

Acoustical analyses were carried out on vocal renditions (Dalla Bella et al., 2007, 2009; Pfordresher et al., 2010). Vowels (e.g., “o” in “sto”) are the targets of acoustical analyses, being the units which carry the maximum of voicing. Each vocal performance was submitted to phonemic segmentation using Praat software (Boersma, 2001) and the EasyAlign tool (Goldman, 2011); vowel onsets and offsets corresponding to the obtained phoneme boundaries were confirmed based on visual inspection of the waveform and of the spectrogram. F0 trajectory within vowels was computed using an accurate autocorrelation method (Boersma, 1993; sampling rate = 100 Hz; Gaussian window = 80 ms). When false pitch detection occurred (i.e., octave jumps) they were manually corrected. Median F0 served as a measure of pitch height. Note onset times and pitch heights served to compute measures of singing proficiency on both the pitch and the time dimensions.

On the pitch dimension, note onset times and pitch heights were used to obtain measures of accuracy (i.e., the distance between the produced pitch or interval and a target) and precision (i.e., the consistency of repeated attempts to produce a pitch or an interval). These measures were treated as independent metrics of singing proficiency. Accuracy and precision were computed separately for absolute pitch, here referring to the absolute pitch height of musical notes, and for relative pitch (i.e., the discrepancy between two subsequent pitches, or interval; for details about these measures, see Berkowska and Dalla Bella, 2009, 2013; Pfordresher et al., 2010; Dalla Bella, 2015a). Accuracy indicates how close is the produced pitch or interval to the target based on the notation. Larger deviation indicates low accuracy. For absolute pitch, accuracy indicates the average difference (in cents, where 1 semitone = 100 cents) between sung and target pitches, regardless of pitch direction (i.e., as to whether the produced pitch was higher or lower than the target). For relative pitch, accuracy refers to the average difference between sung pitch intervals and target intervals. Precision refers to the consistency in the repetition of the same pitch class (for absolute pitch) or of the same interval class (for relative pitch). This measure is obtained by computing how consistently a note or pitch interval deviates from the target across repetitions.

Using distinct metrics for accuracy and precision in terms of absolute and relative pitch has proven in the past as particularly useful for characterizing individual differences in singing skills in the general population (Pfordresher et al., 2010; Berkowska and Dalla Bella, 2013; for discussions, see Dalla Bella et al., 2011; Dalla Bella, 2015a). Indeed, occasional singers may be proficient on one metric while showing poor performance on the other metric (Berkowska and Dalla Bella, 2013). In this study, these measures served to classify occasional singers. For each measure of accuracy and precision, singers were divided into two subgroups (*Accurate* vs. *Less accurate*; *Precise* vs. *Less precise*) by performing a median split.

On the time dimension, two measures were computed from the performances, namely tempo and temporal variability, as done in previous studies (Dalla Bella et al., 2007; Dalla Bella and Berkowska, 2009). Tempo is the mean IOI of the quarter-note. Temporal variability is the coefficient of variation (CV) of the quarter-note IOIs (SD of the IOIs / mean IOI).

### Tapping Tasks

Tapping sequences obtained in the Synchronization Task were pre-processed as follows. The taps corresponding to the first five isochronous tones of each recorded sequence were not analyzed (i.e., there were 30 useful taps). In addition, taps were discarded if they departed by more than the 3 × inter-quartile range from the median inter-tap-interval (ITI) in the trial (i.e., outliers). The remaining taps served to compute synchronization accuracy and consistency.

**Figure 1:**
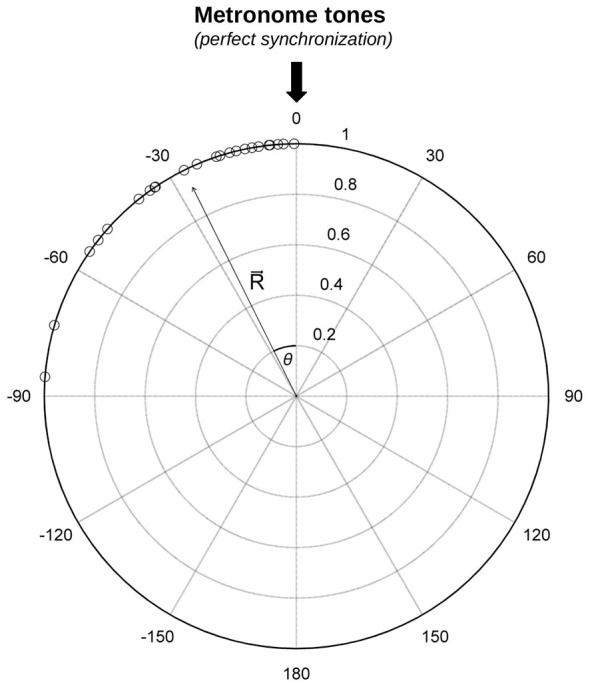
**Example of the distribution of taps from a trial taken from the Synchronization Task (number of useful taps = 30).** The resultant vector *R* and its direction (angle theta, *θ*) are indicated. The angle is a measure of synchronization *accuracy*, the vector length (from 0–1), of synchronization *consistency*.

Synchronization data were analyzed with circular statistics (Fisher, 1995) using the Circular statistics Toolbox for Matlab (Berens, 2009). Circular statistics have been used in the past to analyze synchronization data (e.g., Kirschner and Tomasello, 2009; Pecenka and Keller, 2011; Sowiński and Dalla Bella, 2013) and have an advantage in that they do not require a one-to-one correspondence between taps and pacing stimuli. These statistics are particularly sensitive to individual differences among participants, and thereby are ideally suited to analyze and characterize situations where participants poorly synchronize to the beat (Kirschner and Tomasello, 2009; Sowiński and Dalla Bella, 2013). In circular statistics, the IOI between pacing tones is represented by a circle on a polar scale. One full circle (360 degrees) indicates the IOI between the periodically recurring pacing events. The time of the pacing event corresponds to 0 degrees. Each tap is represented by an angle relative to the time of the pacing event. The distribution of the tap times relative to the pacing stimuli is indicated by dots around the circle (see role plot in Figure 1, for an example). Taps preceding the tone are indicated by negative angles, whereas taps following the tone are represented by positive angles. The angles corresponding to each tapping sequence are transformed into unit vectors, and the mean resultant vector *R* is calculated (Fisher, 1995; Mardia and Jupp, 2000; Berens, 2009). The vector *R* is used to compute synchronization *consistency* and *accuracy* (Sowiński and Dalla Bella, 2013).

**Figure 2:**
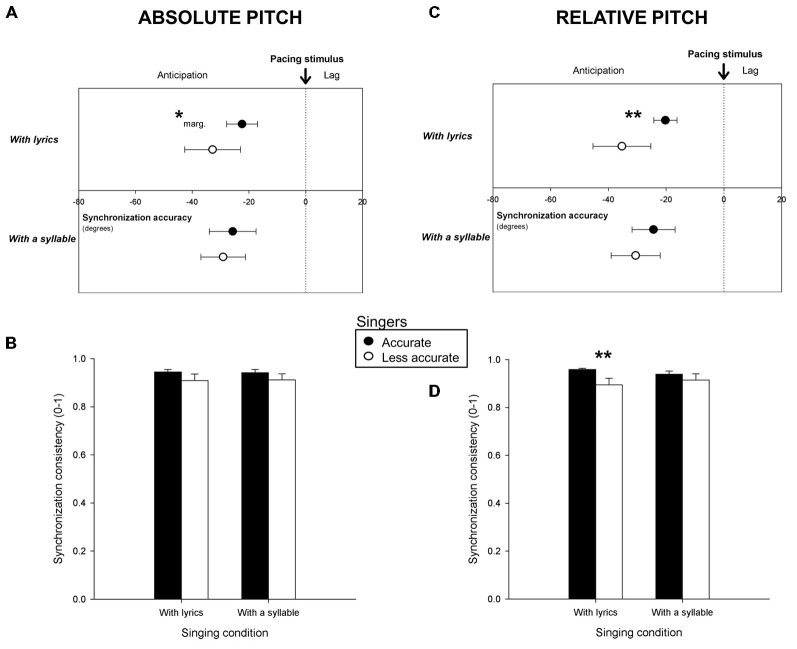
**Mean synchronization accuracy (vector angle, in degrees) and consistency (vector length, from o to 1) for *Accurate* and *Less accuratesingers* in terms of absolute pitch (A,B) and of relative pitch (C,D), when participants sang with lyrics and with a syllable.** **p < or = 0.01, *marg. = marginally significant (*p* = 0.06). Error bars indicate 95% confidence intervals **(A,C)** or Standard Error of the Mean **(B,D)**.

**Figure 3:**
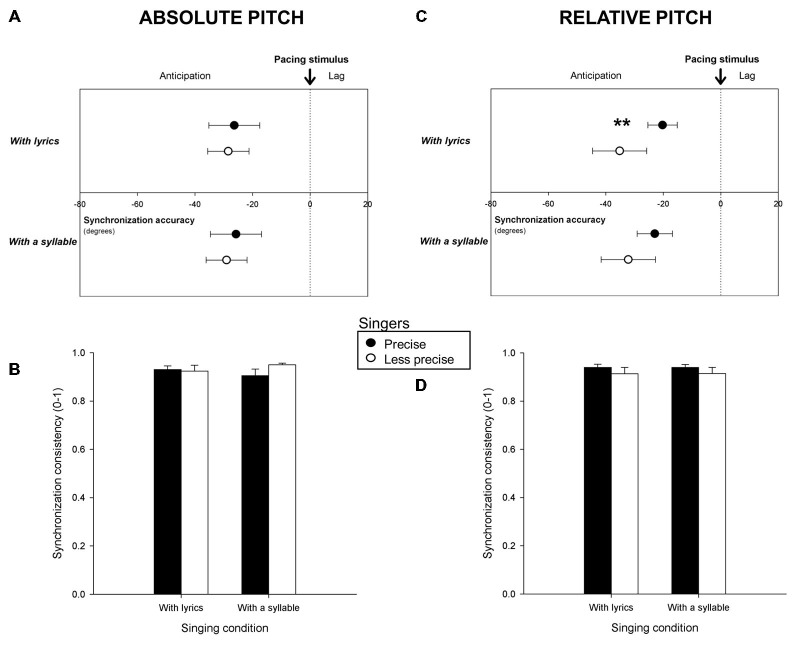
**Mean synchronization accuracy (vector angle, in degrees) and consistency (vector length, from o to 1) for *Precise* and *Less precise singers* in terms of absolute pitch (A,B) and of relative pitch (C,D), when participants sang with lyrics and with a syllable.** **p < or = 0.01. Error bars indicate 95% confidence intervals **(A,C)** or Standard Error of the Mean **(B,D)**.

**Figure 4:**
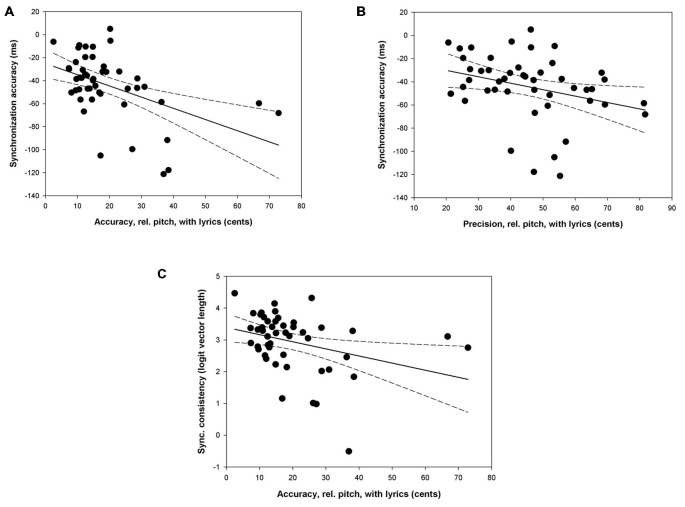
**Scatter plots indicating the relation between singing proficiency for relative pitch when singing with lyrics and synchronization performance. (A,B)** indicate the relation between synchronization accuracy and singing accuracy/precision, respectively. **(C)** illustrates the relation between synchronization consistency and singing accuracy.

Synchronization consistency indicates the variability of the discrepancy between the time of the taps and of the metronome tones. When this discrepancy is constant, consistency is maximal. Consistency corresponds to the length of vector *R* and it ranges between 0 and 1. Zero reflects a random distribution of angles around the circle (i.e., lack of synchronization), whereas a value of one refers to maximum consistency (no variability). Before performing statistical analyses (i.e., *t*-tests or ANOVAs), vector length values were submitted to a logit transformation to reduce data skewness, which is typical of synchronization data (Kirschner and Tomasello, 2009; Sowiński and Dalla Bella, 2013). However, for simplicity, means from untransformed data are reported in the figures (except Figure 2,3,4). Synchronization accuracy is the average difference between the time of the taps and the time of the metronome tones. When a participant taps exactly in correspondence of the tones, accuracy is maximal. This measure corresponds to the angle of the vector *R* (*θ* or relative phase, in degrees). It can be negative or positive, thus indicating whether the participant tapped before or after the pacing events. Data for accuracy were considered exclusively for trials in which participants’ synchronization was above chance, as assessed with the Rayleigh test for circular uniformity (Wilkie, 1983; Fisher, 1995), as done in previous studies (Sowiński and Dalla Bella, 2013). Subgroups of participants were compared using ANOVAs for circular data, namely Watson-Williams two-sample tests (Berens, 2009).

Sequences of taps obtained in the Spontaneous tapping Task were pre-processed by discarding the first 15 and the last 15 taps to avoid practice and fatigue effects. In addition, as done in the Synchronization Task, taps which departed by more than 3 × inter-quartile range from the median ITI in the trial (i.e., outliers) were discarded. The remaining taps were analyzed to obtain measures of the mean ITI and of the variability of the ITIs (the coefficient of variation—CV—of the ITIs, that is the *SD* of the ITIs/ mean ITI). The CV of the ITIs measures the variability in producing regularly repeated time intervals (the smaller the CV of the ITIs, the more accurate the performance). For each participant, the values of mean ITI and CV of the ITIs in the two trials were averaged.

## Results

### Vocal Performance

The Experiment yielded 294 recordings, 147 with lyrics and 147 with a syllable. Subgroups of participants (Accurate vs. Less accurate; Precise vs. Less precise) based on their performance when they sang with lyrics or with a syllable are reported in Table 1 together with measures of singing proficiency on the pitch and on the time dimensions. Significant differences between the subgroups are reported (Bonferroni-corrected *t*-tests). Notably, 77.6% of the participants who were accurate/precise (or less accurate/less precise) in absolute pitch were also classified as such based on relative pitch. Moreover, 73.5% of the participants who were accurate (or less accurate) were also similarly classified as precise (or less precise) singers. As can be seen, differences between all subgroups when participants sang with lyrics and with a syllable were highly significant on the pitch dimension. Renditions did not differ in terms of tempo. However, Less accurate/precise singers in terms of relative pitch were more temporally variable when singing with lyrics than Accurate/Precise singers. This finding is supported by significant correlations between synchronization accuracy/precision and singing temporal variability (*r* = 0.41, *p* < 0.01; *r* = 0.47, *p* = 0.001, respectively). Given this association between synchronization and the time dimension of singing, temporal variability is considered below as a covariate when assessing the link between synchronization skills and singing on the pitch dimension.

**Table 1:** **Singing proficiency in the pitch dimension (accuracy and precision), and in the time dimension (tempo and temporal variability) for Accurate/Precise singers vs. Less accurate/Less precise singers**.

	Singing with lyrics		Singing with a syllable	
Variables	Accurate/Precise singers (*n* = 25) *M (SE)*	Less accurate/Less precise singers (*n* = 24) *M (SE)*	Accurate/Precise singers (*n* = 25) *M (SE)*	Less accurate/Less precise singers (*n* = 24) *M (SE)*
*Classification based on Absolute pitch*
Pitch accuracy (cents)	52.31 (7.82)	296.67 (36.86)***	51.77 (7.34)	273.59 (40.33)***
Tempo (IOI, sec)	0.154 (0.002)	0.150 (0.001)	0.150 (0.002)	0.150 (0.002)
Temporal variability (CV IOI)	0.148 (0.008)	0.168 (0.006)	0.145 (0.014)	0.150 (0.010)

Pitch precision	26.31 (1.58)	55.96 (3.55)***	22.86 (1.46)	50.29 (2.59)***
Tempo	0.153 (0.002)	0.151 (0.002)	0.151 (0.002)	0.149 (0.002)
Temporal variability	0.154 (0.009)	0.162 (0.006)	0.134 (0.008)	0.161 (0.015)
*Classification based on Relative pitch*
Pitch accuracy	11.13 (0.57)	28.40 (3.01)***	9.52 (0.64)	25.77 (3.08)***
Tempo	0.153 (0.002)	0.151 (0.002)	0.152 (0.002)	0.147 (0.002)
Temporal variability	0.140 (0.006)	0.177 (0.007)**	0.136 (0.007)	0.159 (0.015)

Pitch precision	32.90 (1.59)	58.23 (2.09)***	28.35 (1.61)	52.50 (2.68)***
Tempo	0.152 (0.002)	0.152 (0.002)	0.150 (0.002)	0.150 (0.002)
Temporal variability	0.143 (0.007)	0.173 (0.007)*	0.132 (0.008)	0.163 (0.015)

Participants were classified based on absolute and relative pitch performance, and when they sung with lyrics and with a syllable. *p < 0.05, **p < 0.01, ***p < 0.001.

### Tapping Tasks

Participants produced 294 sequences of taps in the Synchronization Task. One percent of the taps (i.e., outliers) was discarded. The remaining taps (29.4, on average, for each trial; *SE* = 1.1 taps) served to compute synchronization accuracy and consistency for the six trials yielded by each participant. Mean synchronization accuracy and consistency for Accurate and Less accurate singers are shown in Figure 2. Figures 2A,B report the results obtained with both groups classified based on absolute pitch, and Figures 2C,D, based on relative pitch. As can be seen in Figures 2A,C both groups tapped prior to the pacing stimuli (i.e., with negative angles significantly different from 0, *p* < 0.05). However, Accurate singers tapped more in the vicinity of the pacing stimuli (i.e., they synchronized more accurately) than Less accurate singers. This effect was visible only for singing with lyrics, when participants were classified based on relative pitch (*F*_(1,47)_ = 8.75, *p* < 0.01) and just failed to reach significance on absolute pitch (*F*_(1,47)_ = 3.79, *p* = 0.06, marginally significant). Further analyses were conducted to assess whether Accurate and Less accurate singers differed in terms of synchronization consistency. Accurate singers were more consistent than Less accurate singers (when singing with lyrics, see Figure 2D) for relative pitch only (*t*_(34.1)_ = 2.72, *p* = 0.01). These differences were confirmed when comparing the 10 most accurate singers (synchronization accuracy = −17.7 degrees with relative pitch; = −19.8 with absolute pitch; synchronization consistency = 0.97 with relative pitch) to the 10 least accurate singers (accuracy = −44.7 degrees with relative pitch, = −34.6 with absolute pitch; consistency = 0.85) in the group (accuracy with lyrics, relative pitch, *F*_(1,18)_ = 16.55, *p* < 0.001; absolute pitch, *F*_(1,18)_ = 4.61, *p* < 0.05; consistency with lyrics, *t*_(12.8)_ = 3.14, *p* < 0.01).

Mean synchronization accuracy and consistency for Precise and Less precise singers are reported in Figure 3. Figures 3A,B indicate the results for singers classified based on absolute pitch measures, Figures 3C,D, based on relative pitch. Precise singers tapped more in the vicinity of the pacing stimuli than Less precise singers when classified based on relative pitch and when singing with lyrics (*F*_(1,47)_ = 8.55, *p* = 0.005). This difference was confirmed when comparing the 10 most precise singers (synchronization accuracy = −19.0 degrees) to the 10 least precise singers (accuracy = −35.9 degrees; *F*_(1,18)_ = 4.57, *p* < 0.05). No more significant differences in synchronization accuracy and consistency between Precise and Less precise singers were found.

The relations between singing proficiency and synchronization showed above when comparing subgroups of singers based on their singing accuracy and precision were further tested with correlational analyses. These analyses were limited to measures of singing accuracy/precision and synchronization accuracy/consistency in the conditions which had shown significant group effects above (see Figure 4)^1^. High singing accuracy and precision for relative pitch when singing with lyrics was associated with high synchronization accuracy (*r* = −0.48, *p* < 0.001; *r* = −0.32, *p* < 0.05; Figures 4A,B respectively), and with high synchronization consistency (*r* = −0.33, *p* < 0.05; Figure 4C)^2^. It can be noted in Figures 4A,C that two participants were particularly inaccurate with values above 60 cents. After removing these outliers the correlations between singing accuracy and synchronization accuracy/consistency remained highly significant (*r* = −0.56, *p* < 0.001; *r* = −0.51, *p* < 0.001), thus confirming that this is a robust finding. Interestingly, singing accuracy and precision are not only associated with synchronization but also with temporal variability (CV IOI) during singing. Accurate and precise singers on the pitch dimensions (in terms of relative pitch) when singing with lyrics were also the least variable on the time dimension, with the lowest CV of the IOIs (*r* = 0.41, *p* < 0.01; *r* = 0.47, *p* < 0.001, respectively). This suggests that the observed relation between singing proficiency and synchronization may be mediated by temporal variability in pitch production. To assess this possibility, partial correlations were carried out to test the relation between singing accuracy/precision and synchronization accuracy/consistency while controlling for temporal variability. Partial correlations revealed that high singing accuracy for relative pitch when singing with lyrics was still associated with high synchronization accuracy (*r* = −0.38, *p* < 0.01). However, the partial correlation between singing precision and synchronization accuracy, and between singing accuracy and synchronization precision failed to reach significance (average *r* = −0.19, *p*s = n.s.).

Additional analyses were conducted to test potential differences between the three melodies used in the study. “Brother John” is a longer melody (including 32 notes) than the other two stimuli (“Jingle bells” and “Sto lat”). Thus, it may be expected that the first melody places heavier demands on singers’ memory than the other two. Separate analyses for each melody, when sung with lyrics and on a syllable, were conducted to check whether the correlations previously identified between singing accuracy/precision and synchronization accuracy/consistency varied as a function of the stimulus. Correlations between singing accuracy in terms of relative pitch (with lyrics) and synchronization accuracy were significant for the three stimuli (“Brother John”, *r* = −0.30, *p* < 0.05; “Jingle Bells”, *r* = −0.31, *p* < 0.05; “Sto Lat”, *r* = −0.49, *p* < 0.001). However, the other two correlations between singing precision and synchronization accuracy, and between singing accuracy and synchronization consistency were significant only for “Brother John” (*r* = −0.30, *p* < 0.05 and *r* = −0.37, *p* = 0.01; for “Jingle Bells”, average *r* = −0.18, *p*s = n.s.; “Sto Lat”, average *r* = −0.17, *p* = n.s.). This finding suggests that memory factors may play a role in the relation between singing proficiency and synchronization.

The Spontaneous tapping Task yielded 96 sequences of taps. 0.6% of the taps (i.e., outliers) were discarded on average. The remaining taps (57.1, on average, for each trial; *SE* = 5.6 taps) were analyzed to calculate the mean ITI and the CV of the ITIs. Accurate/Precise and Less accurate/Less precise singers in terms of absolute and relative pitch did not tap spontaneously at different tempos (with average ITIs of 788 ms and 727 ms, respectively, *p*s = n.s.) and did not differ in terms of temporal variability (with CVs of the ITIs = 0.05 in all groups, *p*s = n.s.). In addition, the participants were not significantly more variable in the Spontaneous tapping Task as compared to the Synchronization Task (CV of the ITIs = 0.04; *t*_(48)_ = 1.38, *p* = n.s.).

## Discussion

The goal of this study was to examine whether singing and synchronization skills are linked in humans. It was found that occasional singers who are particularly accurate and precise at carrying a tune are also very accurate and consistent in moving to the beat of predictable sequences of tones. Accurate singers and Precise singers classified in particular based on relative pitch tapped closer to the beat than did poorer singers. Accurate singers were also more consistent (less variable) than Less accurate singers when tapping to the beat. These differences are visible only when participants sang with lyrics.

Correlational analyses confirmed that singing proficiency covaries with synchronization skills in the chosen sample of occasional singers. Notably, temporal variability during singing is also related to pitch accuracy and precision (i.e., poor singers on the pitch dimension tend also to be poor on the time dimension). Thus, general temporal processing may act as a mediating factor in the relation between pitch production during singing and synchronization to a beat. However, when temporal variability during singing is partialled out, we can still observe a relation between singing and synchronization accuracy. This suggests that temporal variability cannot account alone for the observed link between pitch accuracy and synchronization. Finally, Accurate/Precise singers did not differ from Less accurate/Less precise singers when asked to tap at a spontaneous rate without a pacing stimulus. This finding discards basic motor skills and motivational factors as potential explanations of the observed differences in synchronization skills linked to singing proficiency.

To our knowledge, these findings provide for the first time evidence of a consistent link between singing and synchronization skills. It is noteworthy that this link manifested in specific conditions, namely when participants sung with lyrics and for measures based on relative pitch. That a relation was not observed for all the dimensions and metrics of singing proficiency indicates that the link between singing and synchronization is not due to trivial factors such as general attention or fatigue during the task. More importantly, this observation can be helpful in pinpointing the specific mechanisms for sensorimotor translation common to the two skills. The fact that the link between singing and synchronization emerged only when participants sung with lyrics is particularly intriguing. Occasional singers are typically more accurate and precise on the pitch dimension when they sing with a syllable than with lyrics as we showed in previous studies (Berkowska and Dalla Bella, 2009, 2013; Dalla Bella et al., 2012). This finding is associated to reduced memory load when singing with a syllable, and compatible with evidence that differences in singing abilities are linked to inter-individual variability in working memory and long-term memory (e.g., in poor-pitch singers; Dalla Bella et al., 2009; Tremblay-Champoux et al., 2010). This observation raises the question as to whether memory-related processes may mediate the observed relation between singing and synchronization. Our study was not explicitly set to test this hypothesis. However, item-based analyses showed that the relations between singing and synchronization skills were visible in particular for the longest melody to be imitated (“Brother John”), which was most challenging in terms of memory retrieval. Although we prefer being cautious at this stage, there are indications that memory factors may mediate the relation between singing and synchronization skills. This hypothesis deserves further testing to tease apart the role of memory (working memory and long-term memory) from other factors. Another possible explanation of the link between singing with lyrics and synchronization is that they may both rely on sensorimotor translation mechanisms which are also involved in speech production (Hickok and Poeppel, 2007; Hickok et al., 2011; Tourville and Guenther, 2011). Because singing with lyrics requires the production of text on sustained pitches with a given duration it is likely to engage coupled vocal-speech internal models. There is evidence that phonetic information in spoken and sung sequences similarly improves vocal imitation both on the pitch and timing dimensions (Mantell and Pfordresher, 2013). In addition, vocal imitation of spoken and melodic sequences is comparably accurate in terms of relative pitch, but not of absolute pitch (Mantell and Pfordresher, 2013). That in our study a relation between singing and synchronization was found selectively for relative pitch may be consistent with the activation of vocal-speech related internal models. However, this remains speculative at the moment; the relation between vocal imitation and synchronization to a beat remains to be examined systematically with both speech and music material. In addition, note that other general factors may affect the relation between singing and synchronization to a beat, such as IQ and perceptual skills (e.g., pitch and rhythm perception). For example, although none of the participants showed the typical symptoms of congenital amusia (e.g., difficulty in recognizing familiar melodies; Ayotte et al., 2002), we cannot exclude that variability in perceptual skills affected the relation between singing and synchronization. These factors should be controlled in future studies.

The differences between Accurate/Precise and Less accurate/precise singers were particularly visible for synchronization accuracy, less for consistency. The reason why consistency was not as sensitive to group differences may be that in general all participants were very good synchronizers (with average consistency above 0.9). This degree of consistency in paced tapping to isochronous tones is common in this population without musical training (cf. Sowiński and Dalla Bella, 2013). In the future, synchronization to more complex sequences (e.g., music or amplitude-modulated noise) may provide a measure of consistency more sensitive to individual differences among singers. Accuracy was the most informative indicator of individual differences in singing proficiency. A robust finding attesting that singing proficiency may relate to accuracy in mapping action to perception is that singers showing low singing accuracy and precision tapped earlier than the most proficient singers do. In doing so, they show a consistent bias toward over-anticipating the occurrence of the pacing tone. Tapping before the tones of predictable sequences is a well-known phenomenon in finger tapping (negative mean asynchrony—NMA; e.g., Aschersleben, 2002; Repp, 2005 ; Białuńska et al., 2011; for a review, see Repp and Su, 2013). NMA is linked to the perception of the alignment between a motor response and an external auditory signal. The perceived alignment depends on the mapping at a central cognitive level of the representation of the auditory signal to a correspondent motor plan (e.g., in the Sensory Accumulator Model; Aschersleben et al., 2001; Aschersleben, 2002). In addition, NMA is an indicator of participants’ tendency to anticipate the upcoming tone events and of predictive timing. For example, smaller NMA (greater accuracy) is typically found in musically trained individuals as compared to non-musicians (Repp and Doggett, 2007). The tendency to over-anticipate the occurrence of the beat found in Less accurate/precise singers would indicate a greater predictive motor timing error than in more proficient singers. This interpretation is compatible with recent evidence pointing to differences in auditory-motor translation to account for individual differences in singing, as found in imitation tasks (Pfordresher and Brown, 2007; Berkowska and Dalla Bella, 2009; Hutchins and Peretz, 2012; Pfordresher and Mantell, 2014). Moreover, it is intriguing that a similar bias in a synchronized tapping task has been recently observed in individuals showing speech and motor disorders (i.e., developmental stuttering; Falk et al., 2015). Thus, it is tempting to speculate that a common source of variability in sensorimotor translation may underpin individual differences in vocal production both in the verbal and musical domains.

In sum, there are converging lines of evidence suggesting that sensorimotor translation may be the common denominator to account for individual differences in both singing and synchronization to a beat. This possibility is also consistent with some of current explanations of disorders such as poor-pitch singing (Pfordresher and Brown, 2007; Dalla Bella et al., 2011; Hutchins and Peretz, 2012; Pfordresher et al., 2015a) and beat deafness (Phillips-Silver et al., 2011; Sowiński and Dalla Bella, 2013; Palmer et al., 2014). These conditions may indeed represent the end of a continuum of singing proficiency and synchronization skills, respectively. Even though the majority can carry a tune (Dalla Bella et al., 2007), some individuals, referred to as “poor-pitch singers” or “tone deaf” are inaccurate when asked to sing or imitate a melody (e.g., 10–15% according to previous estimates based on accuracy; Dalla Bella et al., 2007; Pfordresher and Brown, 2007; Dalla Bella and Berkowska, 2009). There is increasing evidence that this condition, at least for a considerable number of poor-pitch singers, may be underpinned by a difficulty to translate perceptual representations into motor plans (Pfordresher and Brown, 2007; Hutchins and Peretz, 2012). Reduced connectivity via the fasciculus arcuatus (i.e., a pathway connecting temporal and frontal brain areas) in poor-pitch singers lends support to this hypothesis (Loui et al., 2009).

Recently, the possibility of a similar mismatch of perception and action has been raised for individuals suffering from beat deafness. This condition indicates self-identified difficulties in tracking or moving to the beat of an external auditory stimulus, such as music or a metronome (Palmer et al., 2014). In particular, two cases have been recently described in our laboratory showing poor synchronization to a beat in the absence of impaired rhythm perception (Sowiński and Dalla Bella, 2013; Dalla Bella and Sowiński, 2015). These findings, reminiscent of the dissociation between perception and action found in poor-pitch singers (Dalla Bella et al., 2007, 2009; Pfordresher and Brown, 2007; Loui et al., 2008; for a review, see Dalla Bella et al., 2011), point to impaired sensorimotor mapping mechanisms in beat deafness. Unfortunately, little is known about singing abilities in beat-deaf individuals. Nevertheless, there is preliminary evidence showing that individuals suffering from congenital amusia (musical defect affecting mostly pitch processing; Ayotte et al., 2002; Peretz and Hyde, 2003), who are typically poor-pitch singers (Dalla Bella et al., 2009) have difficulties in synchronizing to the beat of music (Dalla Bella and Peretz, 2003). In sum, there are indications that malfunctioning sensorimotor translations mechanisms may underpin some cases of poor-pitch singing and poor synchronization in beat-deaf individuals. That the efficiency of sensorimotor translation may account for individual differences in the general unimpaired population in terms of singing proficiency and synchronization to a beat, and at the same time explain music disorders (i.e., extreme cases on a continuum) is particularly appealing. This idea is in keeping with the recent proposal that individual differences in singing proficiency in the general population and poor-pitch singing may stem from the same source (e.g., inverse modeling processes; Pfordresher and Mantell, 2014). Further studies should be devoted to examine both singing proficiency and synchronization to a beat in poor-pitch singers and in beat-deaf individuals.

By showing that accuracy and precision in imitating a song and in tapping to a beat are linked, our findings lend support to the vocal learning and synchronization hypothesis (Patel, 2006, 2008). To our knowledge these findings are the first evidence that synchronization and singing skills are linked in humans, thus supporting previous findings from animal studies linking synchronization to a beat and vocal skills. However, a word of caution is in order. Our study provides mainly correlational evidence of such a link, thus making impossible at the present stage to conclude about the causal role of one of the two skills on the other. For example, at an evolutionary scale, simultaneous signal production may have played a causal role in synchronizing human vocalizations in groups, thus improving their temporal regularity (Bowling et al., 2013). In the future, this hypothesis may be addressed in humans with training studies, by examining the effects of improving synchronization skills with a dedicated program (e.g., rhythmic exercises) on singing proficiency.

## Conflict of Interest Statement

The authors declare that the research was conducted in the absence of any commercial or financial relationships that could be construed as a potential conflict of interest.

## Footnotes

^1^To run standard Pearson correlations, as all data have to be on a linear scale, synchronization accuracy data (angles of vector *R*) were converted into ms (with 360 degrees = 600 ms). Synchronization accuracy in ms instead of angles is reported in the Figure.

^2^The correlations were also run after excluding the three participants who received musical lessons. After removing the participants, the three correlations remained practically unchanged (for singing accuracy—synchronization accuracy, *r* = −0.51, *p* < 0.001; singing precision—sync. accuracy, *r* = −0.31, *p* < 0.05; singing accuracy—sync. consistency, *r* = −0.44, *p* < 0.01). Thus, the observed relation between singing proficiency and synchronization cannot be merely ascribed to experience with music in some participants.
